# Protection of neutrophils by bone marrow mesenchymal stromal cells is enhanced by tumor-associated inflammatory cytokines

**DOI:** 10.3389/fimmu.2024.1361596

**Published:** 2024-04-16

**Authors:** Yingqi Liang, Xiulan Lou, Yazhang Xu, Zhiyuan Zheng

**Affiliations:** ^1^ National Key Laboratory of Macromolecular Drug Development and Manufacturing, School of Pharmaceutical Sciences, Wenzhou Medical University, Wenzhou, Zhejiang, China; ^2^ Gynecology, Second Affiliated Hospital, Zhejiang University School of Medicine, Zhejiang University, Hangzhou, China

**Keywords:** mesenchymal stromal/stem cells, neutrophil, G-CSF, tumor progression, inflammation

## Abstract

Mesenchymal stromal/stem cells (MSCs), which are distributed in many tissues including bone marrow, have been reported to play a critical role in tumor development. While bone marrow, the primary site for hematopoiesis, is important for establishing the immune system, whether MSCs in the bone marrow can promote tumor growth via influencing hematopoiesis remains unclear. We observed that the numbers of MSCs and neutrophils were increased in bone marrow in tumor-bearing mice. Moreover, co-culture assay showed that MSCs strongly protected neutrophils from apoptosis and induced their maturation. G-CSF and GM-CSF have been well-documented to be associated with neutrophil formation. We found a remarkably increased level of G-CSF, but not GM-CSF, in the supernatant of MSCs and the serum of tumor-bearing mice. The G-CSF expression can be enhanced with inflammatory cytokines (IFNγ and TNFα) stimulation. Furthermore, we found that IFNγ and TNFα-treated MSCs enhanced their capability of promoting neutrophil survival and maturation. Our results indicate that MSCs display robustly protective effects on neutrophils to contribute to tumor growth in bone niches.

## Introduction

Mesenchymal stromal cells (MSCs), also known as mesenchymal stem cells, are essential cellular components in many tissues and contribute to tissue repair and homeostasis (1, 2). In addition to giving rise to osteoblasts, chondrocytes, and adipocytes, MSCs also exhibit potent immunosuppressive effects (3, 4). Several studies have demonstrated that MSCs can promote tumor growth and progression via immunosuppressive effects on cells of the adaptive immune system, like T cells (5, 6) and B cells (7–9). MSCs, after their migration to tumor sites, were also shown to promote tumor growth by recruiting monocytes/macrophages (10, 11).

Neutrophils represent the major cellular components in the bone marrow where they proliferate and reach maturation (12, 13). Several studies have demonstrated that neutrophils participate in tumor progression (14–16). However, little is known whether and how MSCs can influence neutrophils during tumor progression. Herein, we examined MSCs and neutrophils in the bone marrow of tumor-bearing mice and tested the effect of MSCs on neutrophils *in vitro*. We found that MSCs protected neutrophils from apoptosis, and promoted neutrophil maturation, probably through producing G-CSF. Moreover, inflammatory cytokines IFNγ and TNFα, which are elevated in the serum of tumor-bearing mice, can strikingly stimulate MSCs to produce more G-CSF. These findings indicate that MSCs can influence neutrophil and may provide the potential therapeutic targets for tumor immunotherapy.

## Materials and methods

### Animals

MMTV-PyMT (FVB/N-Tg (MMTV-PyVT) 634Mul/J) and FVB (FVB/NJ) mice were obtained from the Jackson Laboratory and bred in a specific pathogen-free animal facility of Wenzhou Medical University (temperature 22°C, humidity 59 RH using a 12/12 h dark/light cycle). The animal protocols for the experiments described in this paper were approved by the Ethical Committee of Wenzhou Medical University.

### MSCs isolation and culture

Mouse mesenchymal stromal cells (MSCs) were isolated from the tibia and femur bone marrow aspirates from WT or 8-week-old MMTV-pyMT mice. Cells were cultured in α-MEM medium supplemented with 10% FBS, 2 mM glutamine, 100 U/ml penicillin, and 100 μg/ml streptomycin (all from Invitrogen). Nonadherent cells were removed after 24 hr, and adherent cells were maintained with medium replenishment every 3 days. To obtain MSC clones, cells at confluence were harvested and seeded into 96-well plates by limited dilution. Individual clones were then picked and expanded. Cells were used at 5th to 20th passage. They were identified by markers. CD29, CD44, CD45, Sca1, CD73, and CD11b antibodies and isotype antibodies were purchased from Biolegend (San Diego, CA, USA). MSCs were seeded at a density of 5 × 10^4 cells per well in a 12-well plate, and the supernatant (MSC-CM) was collected after 24 hours.

### RNA-seq gene expression analysis

One mRNA profile GSE164766 was obtained from the Gene Expression Omnibus database (https://www.ncbi.nlm.nih.gov/geo/). This profile was based on the platform of the GPL24247 (Illumina NovaSeq 6000, Mus musculus) and was composed of gene expression of neutrophils in the mammary gland of MMTV-PyMT and PyMT-Cxcr2^-/-^ mice as well as the one of wild-type (WT) bone marrow. The numbers of WT and PyMT mice were both 4. All of the data were freely available online, and this study did not involve medical ethics.

Differential gene expression analysis was performed using the R package DESeq2, and the adjusted P-value and |log2FC| were calculated. Genes that met the cut-off criteria, adjusted P < 0.01 and |log2FC| ≥ 2.0, were considered as differentially expressed genes (DEGs). Kyoto Encyclopedia of Genes and Genomes (KEGG) analysis and gene set enrichment analysis (GSEA) were then performed (17). KEGG terms with a false discovery rate (FDR) < 0.01 were considered statistically significant. For visualization, the R package Pathview was used (18).

### Neutrophil isolation and culture

Single cell suspensions of bone marrow were from 10-week-old WT or MMTV-PyMT mice and neutrophils were isolated using magnetic beads sorting (EasySep™ Mouse Neutrophil Enrichment Kit, Stem Cell, Cat#19762).

### Co-culture neutrophil and MSC

Neutrophils (5 × 10^5^ cells/well) were placed on the upper compartment and MSCs (5 × 10^4^ cells/well) on lower compartment separated by a poly-carbon membrane with 0.4 μm pores and 10 μm thick. After co-culturing for 24 h, neutrophils in the upper compartment were enumerated by flow cytometry. For the immunofluorescence assay, round glass slides were put on the 24 well. MSCs (5 × 10^4^ cells/well) were seeded on the round glass slide in 24 well plate for 24 h, and then neutrophils (5 × 10^5^ cells/well) were added into with or without MSCs well. After co-culturing for 24 h, cells on the round glass slide were stained by Hochest and H3-cit (Red).

### Detection of cytokines

Serum from WT (n=3) and MMTV-PyMT mice (n=3) were obtained and analyzed by ELISA. MSCs were seeded at a density of 5 × 10^4 cells per well in a 12-well plate, and the supernatant (MSC-CM) was collected after 24 hours. The levels of cytokines and chemokines in culture supernatants or serum were assayed by multiplexed bead array immunoassay using Luminex Technology according to the manufacturer’s protocol (Bio-Plex, M60009RDPD). Other ELISA kits: Mouse G-CSF ELISA Kit (Multi Sciences, EK269/2-96).

### Quantitative Real-Time PCR

Total RNA was isolated using RNAprep pure Cell Kit (Feijie Biotech, Shanghai, China). cDNA was reverse-transcribed using the PrimeScript™ RT Master Mix (TaKaRa Biotech, RR036A). The mRNA levels were quantified by real-time PCR (ABI Quant Studio 6, Life) with SYBR Green Master Mix (Thermo Fisher Scientific, USA). The total amount of mRNA was compared with endogenous β-actin mRNA. *β-actin*: forward 5’-CAACGAGCGGTTCCGATG-3’, reverse 5’-GCCACAGGATTCCATACCCA-3’; *G-CSF*: forward 5’-GGCCTAGACCTGAGCAGAAA-3’, reverse 5’-AGGAGGACACAAGAGCCTTC-3’.

### Immunofluorescence

Treated neutrophils were fixed in 4% PFA in PBS for 20 minutes at room temperature and washed with PBS. Nuclei were subsequently stained with Hoechst, and was imaged under a Laser scanning Confocal Microscopy (Leica Biosystems).

### Flow cytometric analysis

Cells were obtained from the tibia and femur bone marrow aspirates from WT and MMTV-PyMT mice, and went through a 70 μm cell strainer, and resuspended in PBS supplemented with 2% FBS. Then cells were suspended in 50 μL staining buffer (PBS containing 2% FBS), with monoclonal antibodies and incubated for 20 minutes at 4°C. Finally, cells were washed twice and resuspended in 200 μL of PBS, and then analyzed on a flow cytometer (Cytoflex, Beckman Coulter). The antibodies used for Flow cytometry as follows: Annexin V-FITC (BD Biosciences, 556547), 7AAD (eBioscience 00-6993-50), PE anti-mouse CD45 (BioLegend 147712), FITC anti-mouse CD11b (eBioscience 11-0112-85), PE anti-mouse Ly6G (eBioscience,12-5931-83), eFluor 450 anti-mouse Lineage (eBioscience 88-7772-72), PE-Cy7 anti-mouse Sca1 (BioLegend 108114), PE anti-mouse CD29 (eBioscience 12-0291-81), FITC anti-CD44 (eBioscience, 11-0441-85), PE anti-mouse CD44 (eBioscience 12-0441-83), PE anti-mouse CD73 (Biolegend 117203).

### Statistical analysis

All data are presented as mean ± SD of the replicates that were derived from several repeats of biological experiments. ns, not significant; *p < 0.05, **p < 0.01, ***p < 0.001, and ****p < 0.0001 by unpaired two-tailed Student’s t-test using the GraphPad Prism software (GraphPad Software, Inc., San Diego, CA, USA).

## Results

### Both MSCs and neutrophils are enriched in bone marrow in tumor-bearing mice

**Figure 1:**
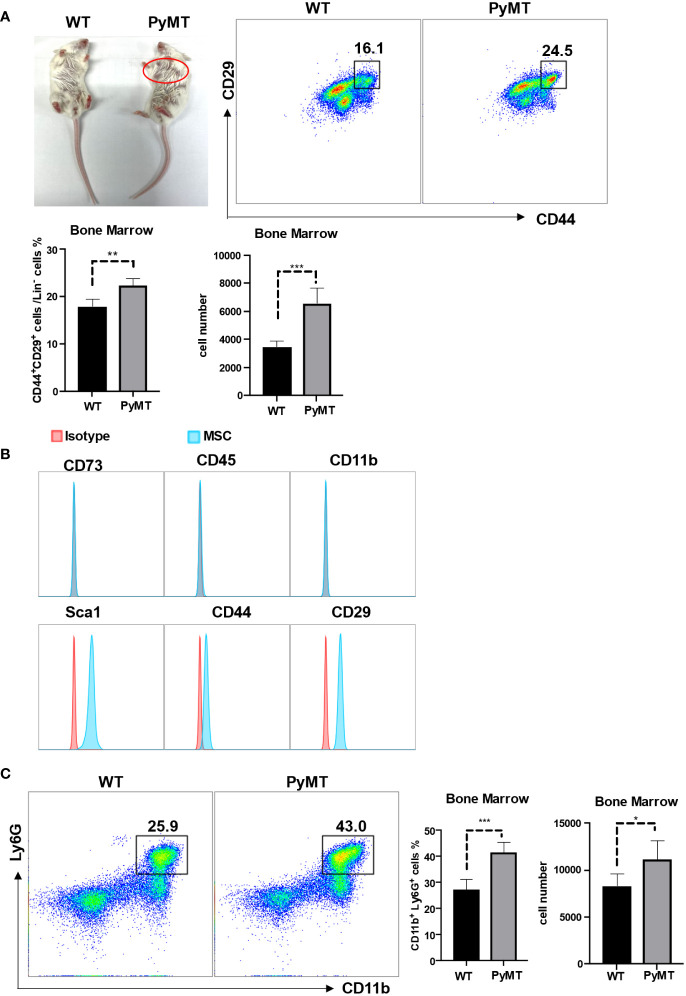
MSCs and neutrophils in bone marrow were increased during tumor progression. **(A)** The phenotype of 10-week-old WT and MMTV-PyMT mice. The red circle shows the tumors. Single-cell suspensions prepared from bone marrow of WT and MMTV-PyMT mice were analyzed for the frequency of Lin^-^CD44^+^CD29^+^ MSCs by flow cytometry after 30 days. n=5. Data are expressed as means ± SD. **(B)** After isolation from bone marrow, MSCs (passage 3) were analyzed for the indicated markers by flow cytometry. **(C)** Single-cell suspensions prepared from bone marrow of WT and MMTV-PyMT mice were analyzed for the frequency of CD11b^+^Ly6G^+^ neutrophils by flow cytometry after 30 days. n=5. Data are expressed as means ± SD. *p < 0.05, **p < 0.01, ***p < 0.001.

To determine whether MSCs in bone marrow may potentially influence neutrophils, we firstly enumerated MSCs and neutrophils in wild-type mice (WT) and tumor bearing mice (10-week-old MMTV-pyMT) in **Figure 1A**. We found that the percentage as well as the absolute number of MSCs were increased in tumor-bearing mice in comparison to control group (**Figure 1A**), indicating that MSCs in bone marrow responded to tumor progression. MSCs from bone marrow were further characterized as CD73^-^CD45^-^CD11b^-^Sca1^+^CD44^+^CD29^+^ (**Figure 1B**). Interestingly, the neutrophils were correspondingly enriched in tumor-bearing mice in comparison to control group (**Figure 1C**). These data indicate that MSCs and neutrophils are both elevated in the bone marrow of tumor-bearing mice.

### Neutrophils derived from PyMT mice displayed stronger proliferative, anti-apoptotic and tumor-promoting capabilities

**Figure 2:**
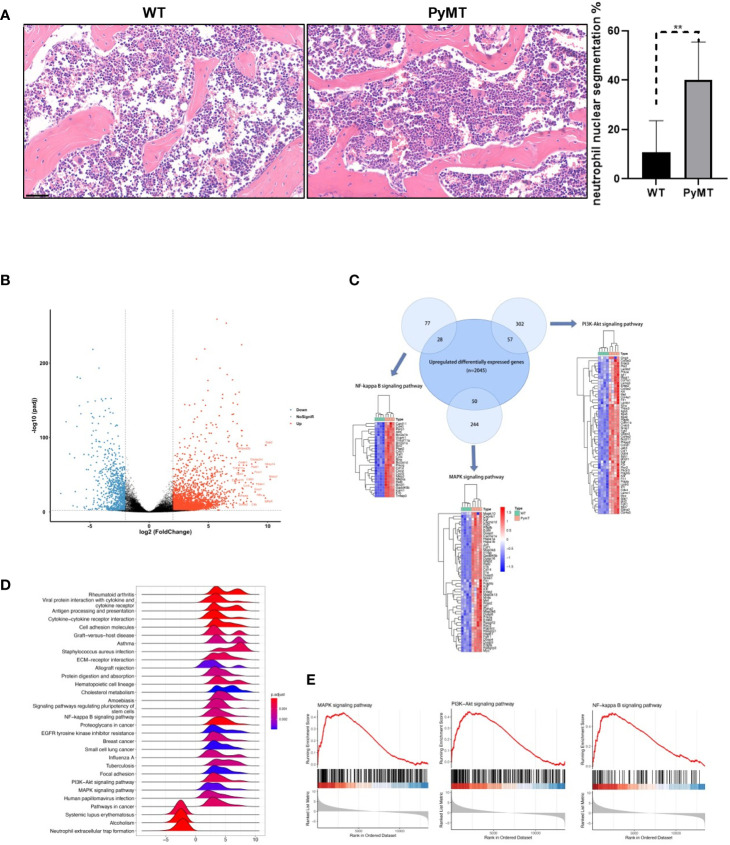
Identifying DEGs between neutrophils derived from WT and PyMT mice. **(A)** H&E staining of bone marrow from WT and PyMT mice. scale bar, 50 µm. **p < 0.01. **(B)** Volcano plot of DEGs between WT and PyMT group. **(C)** KEGG analysis shows that DEGs are enriched in MAPK signaling pathway, NF-kappa B signaling pathway and PI3K-Akt signaling pathway. Venn plot demonstrates the overlapping between DEGs and genes in different pathways. **(D)** GSEA ridgeplot and **(E)** enrichment plots as validation.

Notably, tumor-bearing mice exhibited a noteworthy rise in neutrophil nuclear segmentation compared to wild-type mice (**Figure 2A**), suggesting potential differences in neutrophil characteristics between WT and PyMT mice. To illustrate the difference between neutrophils in the mammary gland of PyMT and WT mice, we analyzed the RNA-seq gene expression from GSE164766. According to DEGs selection criteria |log2FC| ≥ 2, adjusted P-value <0.01, the results showed that 2877 genes (832 significantly down-regulated and 2045 significantly up-regulated genes) differentially expressed between the two groups (**Figure 2B**), using the “DESeq2” package of R software. KEGG analysis showed that changes in gene sets were closely related to MAPK signaling pathway, NF-kappa B signaling pathway and PI3K-Akt signaling pathway (**Figure 2C**). We then performed a GSEA analysis on the 2877 DEGs. Enrichment plots indicated that the gene signatures of three pathways above were significantly enriched in PyMT group (**Figures 2D, E**). Visualization was then achieved through the R package Pathview (**Supplementary Figure 1**). The pathway diagrams showed that gene regulators related to cell cycle, survival and inflammation were up-regulated, suggesting neutrophils derived from tumor-bearing mice might have stronger capabilities of proliferation, anti-apoptosis and tumor-promotion.

### MSCs protected neutrophils from apoptosis and promoted neutrophil maturation

**Figure 3:**
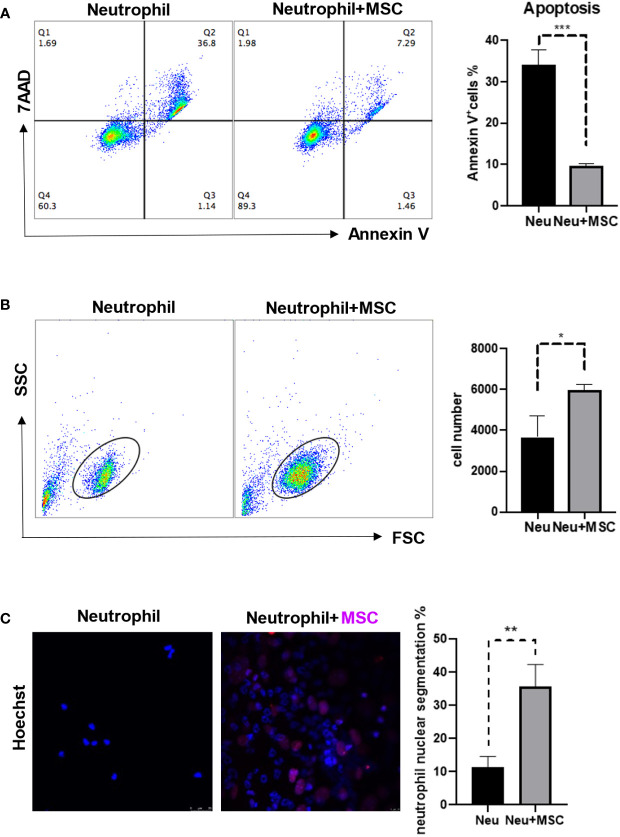
MSCs reduced the neutrophil apoptosis and promoted neutrophil maturation. **(A, B)** MSCs (5 × 10^4^ cells/well) and neutrophils (5 × 10^5^ cells/well) were co-cultured by the transwell assay for 24 h before the apoptosis ratios were analyzed by flow cytometry. Neutrophils were obtained and stained by Annexin V and 7AAD. Data are expressed as means ± SD. **(C)** MSCs and neutrophils were co-cultured for 24 h, and then were determined by Immunofluorescence; MSC nuclei were stained by H3-cit (Red). scale bar, 25 µm. *p < 0.05, **p < 0.01, ***p < 0.001.

To explore the possible influence of MSCs on neutrophils, we isolated neutrophils and MSCs from bone marrow respectively and co-cultured them for 24 h. Interestingly, Annexin V^+^ neutrophils, reflecting cells undergoing apoptosis, were significantly reduced when co-cultured with MSCs (**Figure 3A**). Consistently, there were more remaining neutrophils in the co-culture (**Figure 3B**). In addition, the neutrophils are more mature, as reflected by the increased segmentation of the nuclei, in the co-culture (**Figure 3C**). These data demonstrate that MSCs may act as protectors of neutrophils in bone marrow.

### G-CSF played the key role in MSCs-neutrophils axis

**Figure 4:**
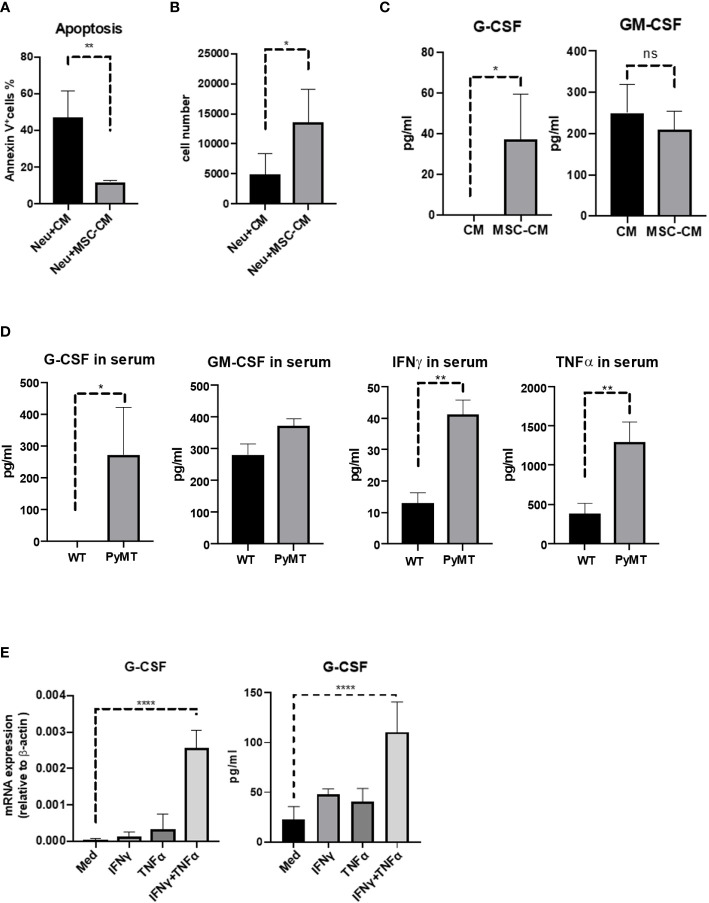
TNFα and IFNγ induced MSCs to produce more G-CSF. **(A, B)** Neutrophils (5 × 10^5^ cells/well) were treated with or without MSC-CM for 24 h before the apoptosis ratios were analyzed by flow cytometry. Neutrophils were obtained and stained by Annexin V and 7AAD. Data are expressed as means ± SD. **(C)** MSCs (5 × 10^4^ cells/well) were seed in 12-well plate and obtained the supernatant (MSC-CM) after 24 h. And then control group (CM) and MSC-CM were detected by ELISA. n=3, data are expressed as means ± SD. **(D)** Serum from WT and MMTV-PyMT mice were obtained and analyzed by ELISA. n=3, data are expressed as means ± SD. **(E)** MSCs (5 × 10^4^ cells/well) were treated with IFNγ (10 ng/mL), TNFα (10 ng/mL), or IFNγ (10 ng/mL) combined with TNFα (10 ng/mL) for 24 h, respectively. And then cells were detected the mRNA level of G-CSF. MSCs (5 × 10^4^ cells/well) were treated with IFNγ (10 ng/mL), TNFα (10 ng/mL), or IFNγ (10 ng/mL) combined with TNFα (10 ng/mL) for 24 h, respectively. And then obtained the supernatant and detected by ELISA. Data are expressed as means ± SD. *p < 0.05, **p < 0.01, ***p < 0.001, ns, no significant.

We next determined whether the protective effect of MSCs on neutrophils was mediated by soluble factors produced by MSCs. To this end, we cultured neutrophils with or without the media conditioned by MSCs (MSC-CM). Compared with control group, MSC-CM reduced neutrophil apoptosis and prevented the loss of neutrophils (**Figures 4A, B**), suggesting that certain cytokines derived from MSCs may mediate the protective effect. G-CSF and GM-CSF are used widely to promote the production of neutrophils (19). Therefore, we examined the levels of G-CSF and GM-CSF in media conditioned by MSCs and found that only G-CSF was elevated in the supernatant of MSCs, in comparison to control group which was undetectable (**Figure 4C**). Furthermore, we found that G-CSF level was higher in the serum of tumor-bearing mice but undetectable in the WT group (**Figure 4D**). Interestingly, the levels of IFNγ and TNFα, which are critical inflammatory cytokines, were also remarkably increased in tumor-bearing mice (**Figure 4D**). To test whether the tumor-associated IFNγ and TNFα can stimulate the production of G-CSF, we treated MSCs with the two cytokines. MSCs treated with IFNγ and TNFα in combination were found to produce much more G-CSF than the MSCs untreated or treated with a single cytokine both at mRNA level and protein level (**Figure 4E**), indicating that MSCs may exert their protective effects on neutrophils via producing G-CSF and that the G-CSF expression in MSCs is further upregulated under inflammatory conditions that are characteristic of tumor microenvironment.

### IFNγ and TNFα-treated MSCs possess a stronger protective effect on neutrophils

**Figure 5:**
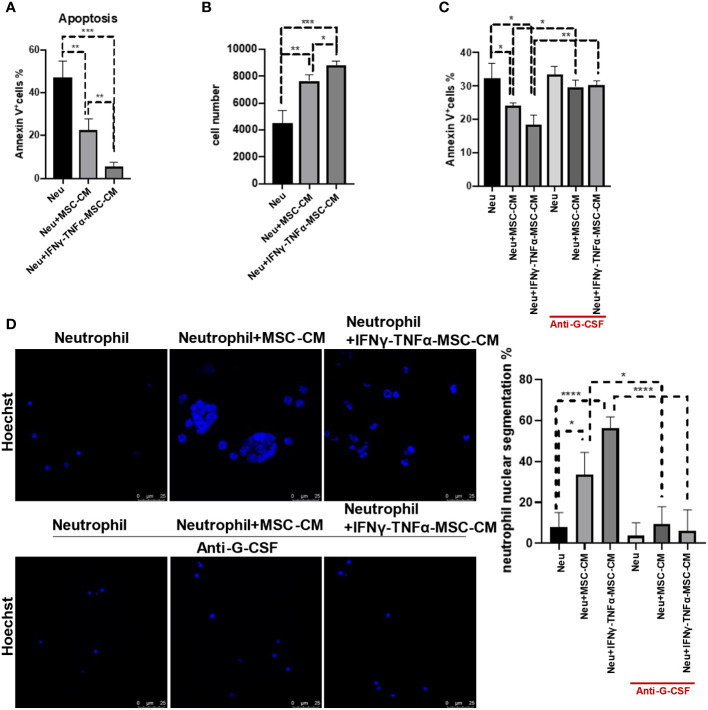
MSCs treated with IFNγ and TNFα displayed the stronger ability of reducing neutrophil apoptosis and promoting neutrophil maturation. **(A, B)** MSCs (5 × 10^4^ cells/well) were treated with IFNγ (10 ng/mL) combined with TNFα (10 ng/mL) for 24 h, respectively. And then we washed the cells and cultured for another 24 h to collect the conditional media (MSC-CM). Neutrophils (5 × 10^5^ cells/well) treated with or without MSC-CM for 24 h before the apoptosis ratios were analyzed by flow cytometry. Control group is neutrophils with no treatment. Data are expressed as means ± SD. **(C)** Neutrophils were cultured at a density of 5 × 10^5 cells per well, treated either with or without MSC-CM, and supplemented with or without anti-G-CSF (10 μg/mL) for 24 h before analyzing apoptosis ratios using flow cytometry. Data are expressed as means ± SD. **(D)** Neutrophils were cultured at a density of 5 × 10^5 cells per well, treated either with or without MSC-CM, and supplemented with or without anti-G-CSF (10 μg/mL) for 24 h, and then were determined by Immunofluorescence; scale bar, 25 µm. *p < 0.05, **p < 0.01, ***p < 0.001.

Next, we determined whether IFNγ and TNFα-treated MSCs can protect neutrophil more effectively. MSCs treated with IFNγ and TNFα indeed displayed an increased capability to reduce neutrophil apoptosis and to sustain the number of neutrophils (**Figures 5A, B**). Blockage of G-CSF by antibody could attenuate the protective effect on neutrophils (**Figure 5C**). Moreover, more hypersegmented nuclei were observed in the neutrophils that had been exposed to IFNγ and TNFα-treated MSCs, but it had no such an effect when G-CSF signaling was abolished using antibody (**Figure 5D**). These data demonstrate that IFNγ and TNFα-induced MSCs can further protect neutrophils and promote their maturation via G-CSF signaling.

## Discussion

Numerous studies have demonstrated that MSCs can migrate into tumor microenvironment and contribute to tumor progression (11, 20, 21). In irradiated mice that were subsequently reconstituted through intra-bone injection of GFP^+^ bone marrow cells, it was shown that the bone marrow MSCs are actively recruited to tumors (22). And the previous work found that MSCs could remodel the pre-metastatic niche via crosstalk with neutrophils (23). However, there are few studies that address the function of MSCs in bone marrow during tumor development. Our results showed that bone marrow MSCs can protect neutrophils from apoptosis and promote their differentiation into hypersegmented neutrophils *in vitro*, suggesting that MSCs may play a critical role in neutrophil formation in bone marrow.

Neutrophils accumulate in the tumor microenvironment or metastatic niche to promote tumor progression. How neutrophils are mobilized and recruited to tumor was addressed in several studies. Single-cell analysis of bone marrow revealed breast cancer can remotely induce a myeloid bias on hematopoietic stem cells by reprogramming the bone marrow microenvironment (24). However, there is a scarcity of studies exploring the role and features of neutrophils within the bone marrow throughout tumor progression. Our investigation revealed that neutrophils derived from tumor-bearing mice exhibited increased neutrophil nuclear segmentation along with heightened proliferation, anti-apoptotic tendencies, and promotion of tumor growth.

Inflammation is one of the major hallmarks of cancer (25). Most of the studies have focused on the interaction between tumors and inflammatory cells and the effects of inflammatory cytokines on tumor development (26–28), the effects of MSCs on hematopoietic cells in bone marrow during tumor progression have been rarely explored. We found that the levels of inflammatory cytokines, such as IFNγ and TNFα, were increased in tumor-bearing mice. Furthermore, bone marrow-derived MSCs could gain increased protective effects on neutrophils when they were treated with IFNγ and TNFα. These data indicate that MSCs in bone marrow could play an increasingly protective effect on neutrophils with increased inflammation associated with tumor progression.

G-CSF and GM-CSF are widely used to promote the production of granulocytes (19). G-CSF drives granulocytic cells to undergo morphological changes, such as the acquisition of primary and specific granules, and nuclear condensation during neutrophil maturation (19). We identified MSCs as a cellular source of G-CSF and its production by MSCs can be elevated by IFNγ and TNFα treatment. These data suggest that MSCs can produce more G-CSF to enrich neutrophils under inflammatory condition. Together, these results provide insights into the potential function of MSCs in bone marrow during inflammation and tumorigenesis.

## Data availability statement

The original contributions presented in the study are included in the article/**Supplementary Material**. Further inquiries can be directed to the corresponding author.

## Ethics statement

The animal protocols for the experiments described in this paper were approved by the Ethical Committee of Wenzhou Medical University. The study was conducted in accordance with the local legislation and institutional requirements.

## Author contributions

YL: Data curation, Formal Analysis, Investigation, Methodology, Validation, Writing – original draft, Writing – review & editing. XL: Data curation, Formal Analysis, Methodology, Writing – original draft, Writing – review & editing. YX: Data curation, Formal Analysis, Methodology, Writing – original draft, Writing – review & editing. ZZ: Conceptualization, Funding acquisition, Project administration, Supervision, Validation, Writing – original draft, Writing – review & editing.

## Supplementary material

Supplementary Figure 1 Pathway analysis of DEGs signature in different pathways. The 2877 DEGs have strong positive correlations with gene regulators that activate MAPK signaling pathway, NF-kappa B signaling pathway and PI3K-Akt signaling pathway.
