# Supplementary material for: Protection of neutrophils by bone marrow mesenchymal stromal cells is enhanced by tumor-associated inflammatory cytokines
Source: Front Immunol. 2024 Apr 16;15:1361596. doi: 10.3389/fimmu.2024.1361596 (PMC11058655; doi:10.3389/fimmu.2024.1361596)
Supplement: Supplementary Figure 1 — Pathway analysis of DEGs signature in different pathways. The 2877 DEGs have strong positive correlations with gene regulators that activate MAPK signaling pathway, NF-kappa B signaling pathway and PI3K-Akt signaling pathway. [file DataSheet_1.docx]

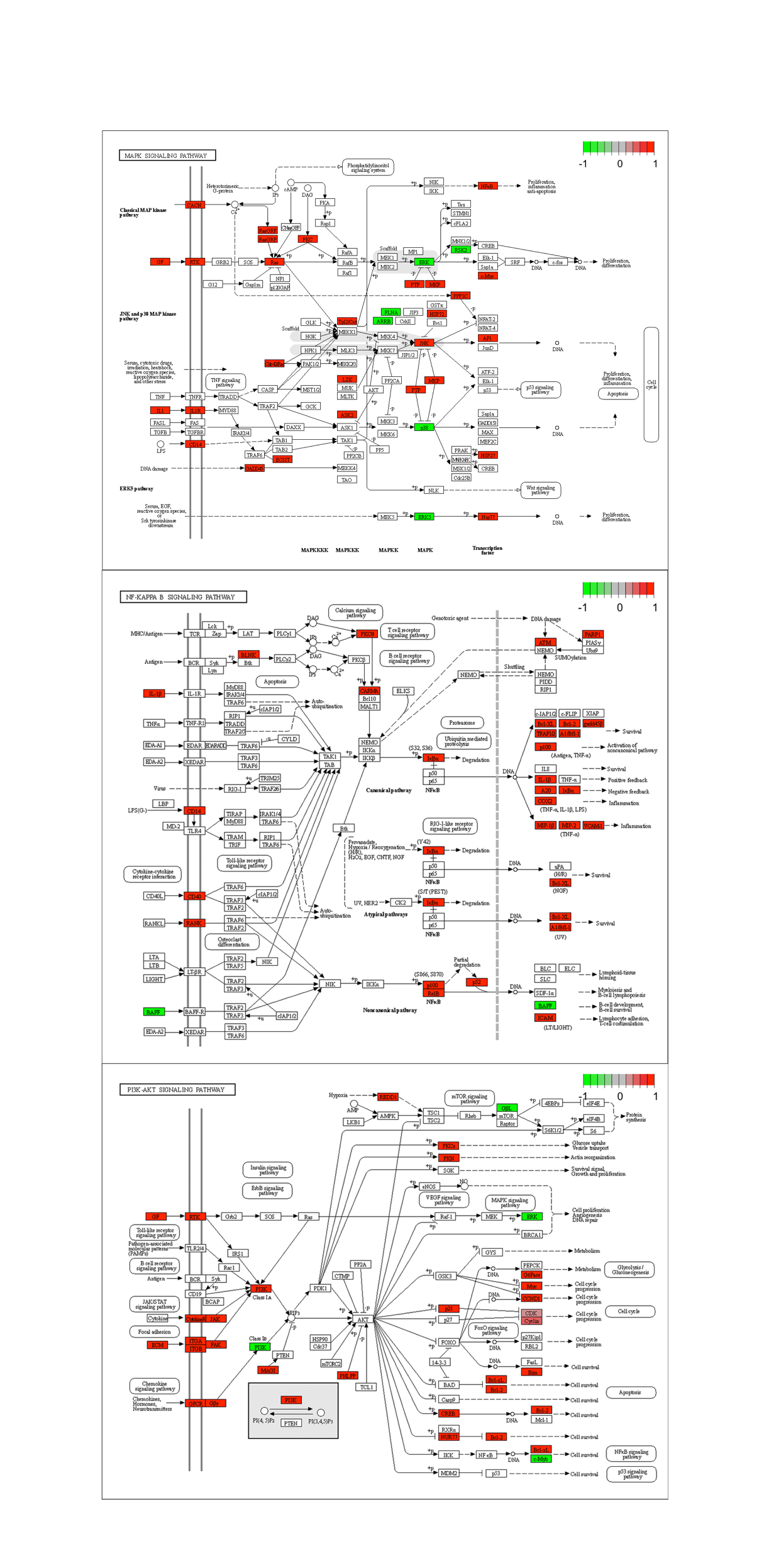


**Supplementary figure 1. Pathway analysis of DEGs signature in different pathways.** The 2877 DEGs have strong positive correlations with gene regulators that activate MAPK signaling pathway, NF-kappa B signaling pathway and PI3K-Akt signaling pathway.
